# Ivermectin: A Controversial Focal Point during the COVID-19 Pandemic

**DOI:** 10.3390/life12091384

**Published:** 2022-09-06

**Authors:** Manuel Castillejos-López, Luz Maria Torres-Espíndola, Juan Carlos Huerta-Cruz, Edgar Flores-Soto, Bianca S. Romero-Martinez, Rafael Velázquez-Cruz, Anjarath Higuera-Iglesias, Ángel Camarena, Ana Karen Torres-Soria, Citlaltepetl Salinas-Lara, Rosario Fernández-Plata, Noé Alvarado-Vásquez, Héctor Solís-Chagoyán, Víctor Ruiz, Arnoldo Aquino-Gálvez

**Affiliations:** 1 Departamento de Epidemiología y Estadística, Instituto Nacional de Enfermedades Respiratorias Ismael Cosío Villegas, Mexico City 14080, Mexico; 2 Laboratorio de Farmacología, Instituto Nacional de Pediatría, Mexico City 04530, Mexico; 3 Unidad de Investigación en Farmacología, Instituto Nacional de Enfermedades Respiratorias Ismael Cosio Villegas, Mexico City 14080, Mexico; 4 Departamento de Farmacología, Facultad de Medicina, Universidad Nacional Autónoma de México, Mexico City 04510, Mexico; 5 Laboratorio de Genómica del Metabolismo Óseo, Instituto Nacional de Medicina Genómica, Mexico City 14610, Mexico; 6 Departamento de Investigación en Epidemiología Clínica, Instituto Nacional de Enfermedades Respiratorias Ismael Cosío Villegas, Mexico City 14080, Mexico; 7 Laboratorio de HLA, Instituto Nacional de Enfermedades Respiratorias Ismael Cosío Villegas, Mexico City 14080, Mexico; 8 Red MEDICI, Carrera de Médico Cirujano, Facultad de Estudios Superiores de Iztacala UNAM, Mexico City 54090, Mexico; 9 Departamento de Bioquímica, Instituto Nacional de Enfermedades Respiratorias Ismael Cosio Villegas, Mexico City 14080, Mexico; 10 Subdirección de Investigaciones Clínicas, Instituto Nacional de Psiquiatría Ramón de la Fuente Muñiz, Mexico City 14370, Mexico; 11 Laboratorio de Biología Molecular, Departamento de Fibrosis Pulmonar, Instituto Nacional de Enfermedades Respiratorias Ismael Cosío Villegas, Mexico City 14080, Mexico

**Keywords:** SARS-CoV-2, ivermectin, antiviral

## Abstract

The SARS-CoV-2 pandemic has confirmed the apocalyptic predictions that virologists have been making for several decades. The challenge the world is facing is that of trying to find a possible treatment, and a viable and expedient option for addressing this challenge is the repurposing of drugs. However, in some cases, although these drugs are approved for use in humans, the mechanisms of action involved are unknown. In this sense, to justify its therapeutic application to a new disease, it is ideal, but not necessary, to know the basic mechanisms of action involved in a drug’s biological effects. This review compiled the available information regarding the various effects attributed to Ivermectin. The controversy over its use for the treatment of COVID-19 is demonstrated by this report that considers the proposal unfeasible because the therapeutic doses proposed to achieve this effect cannot be achieved. However, due to the urgent need to find a treatment, an exhaustive and impartial review is necessary in order to integrate the knowledge that exists, to date, of the possible mechanisms through which the treatment may be helpful in defining safe doses and schedules of Ivermectin.

## 1. Introduction

Ivermectin (IVM) is a broad-spectrum antiparasitic agent, developed and funded by Merck & Co. in 1974 to control and eradicate onchocerciasis caused by the parasitic worm Onchocerca volvulus in West Africa, which in the 1980s infected approximately 340,000 people [1,2]. At the time, Africa did not have the resources necessary to seek treatments for this condition. The avermectins, of which IVM is a member, were discovered by Professor Satoshi Ōmura as fermentation products of the bacterium Streptomyces avermitilis at the Kitasato Institute in Tokyo. For this discovery, he received the 2015 Nobel Prize in Physiology and Medicine, which he shared with William Campbell. IVM is used to treat onchocerciasis, lymphatic filariasis, strongyloidiasis and scabies, and, very recently, has been used to combat lice. The drug’s low cost, high efficacy, safety, and marked tropism for helminths, as well as the fact that it has almost no impact on human biochemistry, have led to the inclusion of IVM in the twentieth list of essential medicines and sixth list of vital medicines in children, a recommendation made by the expert committee of the World Health Organization (WHO) in 2019 [2,3,4]. The safety profile is attributed to its selective affinity for ion channels [5,6].

In humans, the SARS-CoV-2 virus is transmitted through aerosols produced by infected people by talking, coughing or sneezing [7]. SARS-CoV-2 has four structural proteins: spike glycoprotein (S), small envelope glycoprotein (E), membrane glycoprotein (M), and nucleocapsid protein (N) [8,9]. Once in the respiratory tract or oral mucosa, it binds to the angiotensin-converting enzyme 2 (ACE2) receptor to enter the cells. This process is mediated by the proteolytic cleavage of the S protein’s receptor binding domain (RBD) by the transmembrane protease serine 2 (TMPRSS2) [10]. This receptor is abundantly expressed in various tissues, mainly in enterocytes, renal tubules, gallbladder, cardiomyocytes, the cells of the reproductive organs, placentals, trophoblasts, ductal cells, the eyes, vasculature [11,12], lung epithelium [13] and mucosa of the oral cavity [14]. The tissue location of the receptors is relevant, since, in this way, the clinical manifestations of the infection can be explained. Common early symptoms are fever, a cough, headache, chest tightness, dyspnea and myalgias or fatigue, amongst other symptoms [15,16]. The course of the disease is influenced by various factors that determine its severity, such as age, sex [17], comorbidities and genetics, which contribute to the development and evolution of the infection. Considering these and other aspects, there is a wide range of clinical presentations. Young people mostly experience a mild illness, and only a tiny percentage of cases are under 19 years of age [18], which, in part, can be attributed to the body’s capacity to modulate an appropriate balance of the pro-inflammatory and anti-inflammatory responses, which diminishes with aging [17,19]. Moreover, sexual differences have been observed, with men having a higher risk than women of progressing to severe disease [19,20]. The most frequently reported comorbidities worldwide are obesity, hypertension and cardiovascular disease, posing a risk of severity and death [21]. One of the risk factors usually overlooked is the socioeconomic level, posing a greater risk of severity and death [22,23]. In some countries, such as the United States, minority populations have between a 21% and 35% higher probability of being hospitalized than Caucasian populations, and this increase is often associated with other comorbidities [23]. The same situation has been observed in the UK, where minority populations are at higher risk of adverse outcomes than the Caucasian population [24]. Furthermore, minority patients were primarily young and/or overweight/obese and had type-2 diabetes, hypertension, or asthma. Moreover, they also lived in disadvantaged areas compared to white patients [23]. One study reported that patients with high SOFA, qSOFA, APACHE II and SIRS scores, who also had some subsets of lowered immune cells, elevated inflammatory indices, dysregulated multi-organ damage biomarkers, and deleterious complications, were at increased risk of hospital death from COVID-19 [25]. SARS-CoV-2 infection, in severe cases, causes multisystemic inflammation, which affects multiple organs, including the lungs, heart, kidneys, liver and pancreas, and also causes endothelial damage, often leading to death or complications [26].

**Figure 1:**
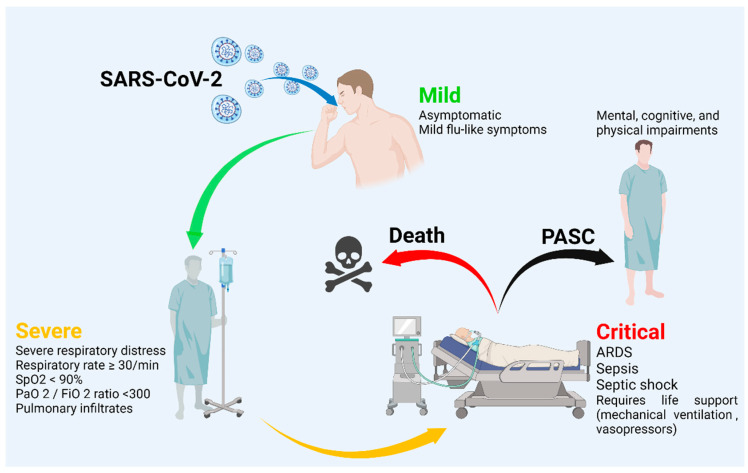
COVID-19 severity classification. Created with BioRender.com.

It is noteworthy that, even though COVID-19 is described as an acute infectious disease, recently, there has been a rise in post-acute symptoms, referred to as the post-acute sequelae of SARS-CoV-2 infection (PASC). In Italy, France and the United States, around 66% to 87% of hospitalized patients remain symptomatic after being discharged, especially those with severe cases of COVID-19. The clinical manifestations presented in PASC vary greatly, affecting multiple systems and causing mental, cognitive and physical impairments [27] (Figure 1).

COVID-19 is an infectious disease producing mild symptoms in most cases [28]. The Chinese Center for Disease Control initially described the different clinical spectra of the disease, which they were classified as mild, severe and critical. Asymptomatic patients may present with mild or no pneumonia and, despite having no symptoms, asymptomatic patients are a potential source of infection [29], while mild cases sometimes present as an influenza-like illness. Some of these cases can progress to severe cases, where hospitalization is required, as well as intensive therapy that includes non-invasive and invasive ventilation, along with antipyretics, antivirals, antibiotics and steroids [29]. Severe cases present with dyspnea, a respiratory rate of ≥30/min, a blood oxygen saturation of ≤93%, a PaO_2_/FiO_2_ ratio of <300 and pulmonary infiltrates, resulting in respiratory failure [29]. The WHO defines the severity of the disease as follows. A critical case is defined by the criteria of acute respiratory distress syndrome (ARDS), sepsis, septic shock or other conditions that would generally require the provision of life support therapies, such as mechanical ventilation (invasive or non-invasive) or vasopressor therapy [16]. A severe case is defined by any of the following parameters: oxygen saturation of <90% in ambient air, signs of severe respiratory distress (the use of accessory muscles, inability to complete sentences and, in children, the very severe retraction of the chest wall, grunting, central cyanosis or the presence of any other general signs of danger) (Figure 1). A non-severe case is defined as the absence of signs of severe or critical COVID-19 [30]. Given the critical importance of the cytokine storm in the pathophysiology of SARS-CoV-2, this has become a major pharmacological target during the process of drug discovery, and many proposed treatments have immunomodulatory and anti-inflammatory effects [8,31].

The innate immune cells (macrophages, dendritic cells and circulating monocytes) can be activated by pathogens through the TLR (toll-like receptor) by the recognition of PAMPs (pathogen-associated molecular patterns). In the case of viruses, these are called VAMPs (viral-associated molecular patterns), and this signaling pathway has been associated with the immune response induced by the SARS-CoV-2 virus [8,32,33]. A major player in the immunopathogenesis of COVID-19 is the “cytokine storm”, which can lead to pulmonary dysfunction, multiorgan failure and death. This response can be induced by the TLR–RNA interaction. Through in silico studies, the TLR3, -7 and -9 have shown a strong binding affinity for the SARS-CoV-2 mRNAs that encode for NSP10, E-protein, NSP8 and S8, leading to a pro-inflammatory response [34]. Specifically, the TLR4–S protein interaction promotes the expression of the ACE2 receptor, facilitating viral entry, and it has been linked to a pro-inflammatory and hypercoagulatory state in COVID-19 patients [35].

After TLRs and other pattern recognition receptors (PRR) are activated by the SARS-CoV-2 virus, the activation of the interferon regulatory factors (IRF3 and IRF7) and the nuclear transcription factor (NF-κβ) takes place. NF-κβ mediates the production of pro-inflammatory cytokines (TNF-α, IL-1 and IL-6), while IRF3 and IRF7 stimulate the production of type-I and -III interferons (IFN-α, -β and -λ). IFNs activate the JAK/STAT signaling cascade that, in turn, activates the synthesis of the pro-inflammatory cytokines (TGF-β, IL-2, IL-4, IL-6 and IL-12) [8,36].

Evidence suggests that the cytokine storm is a determining factor in the death of critically ill patients because it triggers an exaggerated systemic inflammatory response that leads to tissue damage [37,38]. This storm occurs when the leukocytes are activated and release pro-inflammatory cytokines, such as IL2, IL7, GSCF, IP10, MCP1, MIP1A, IL-6, IL-10, IL-8, TNF-α, IL-1β, IL-2R and other pro-inflammatory markers, such as ferritin, hs-CRP and procalcitonin. Exacerbated increases in these cytokines have been observed in patients who died from COVID-19 [15,26]. The most common form of organ failure in critical illness due to COVID-19 is acute hypoxemic respiratory failure, which clinically presents as acute respiratory distress syndrome (ARDS) [31,39]. This syndrome includes severe pulmonary infiltration/edema and inflammation, leading to impaired alveolar homeostasis; impaired lung physiology, resulting in pulmonary fibrosis; endothelial inflammation; vascular thrombosis; and immune cell activation [40]. Lymphocytopenia occurs in a high percentage of patients upon admission (83.2%) [41]. However, the specific conditions that cause this decrease in the number in lymphocytes are yet to be determined, as are the roles of participating factors related to the host or virus.

Conceivably, the necessity of effective pharmacological treatments directed against the COVID-19 disease has led to the investigation of the application of known drugs and their possible use in these patients. The multifactorial characteristics of COVID-19 have encouraged the development of different strategies for upgrading the clinical treatment of the disease. We and other research groups have taken a particular interest in IVM in this case, given its mechanisms of action and favorable safety profile. Multiple studies have sought to determine IVM’s effects on the pathophysiology of SARS-CoV-2. However, its effectiveness must be defined with certainty through well-designed clinical trials.

## 2. Mechanisms of Action of Ivermectin

Ivermectin is a broad-spectrum drug with numerous effects on parasites, nematodes, arthropods, flavivirus, mycobacteria and mammals through a variety of mechanisms. The mechanism of action of Ivermectin as an anthelmintic agent at various stages in the life cycle works by binding to glutamate-gated chloride ion channels in the nerve cells and invertebrate muscles of microfilaria [42,43]. The union of IVM with the channels causes an increment in the cell membrane permeability for chloride ions, hyperpolarizing the membrane and interrupting the motility, feeding and reproduction, leading to the paralysis and death of the parasite. In addition to the glutamate-gated ion channels, ivermectin is also an agonist of the neurotransmitter gamma-aminobutyric acid (GABA)-activated channels. Since GABA channels in mammals are exclusively found in the central nervous system, and IVM does not readily cross the blood–brain barrier, it has a favorable safety profile in regard to the treatment doses used in humans [6,42,43]. Ivermectin achieves adequate levels of availability when administered orally, and due to its high lipid solubility, IVM is widely distributed, with a volume of distribution of 46.8 L. The metabolism of IVM is hepatic, primarily effected by the CYP3A4, and it is removed through the feces and only 1% through urine [2,42,44].

## 3. Possible Benefits of Ivermectin in SARS-CoV-2

The following sections will review the effects attributed to IVM derived from some in vivo and in vitro studies, beginning with one of the most controversial effects.

### 3.1. Antiviral Activity

One of the most exciting effects of IVM is its possible role as an antiviral against COVID-19 [45]. Certain reviews have emphasized the antiviral effect of IVM in vitro and in vivo against RNA and DNA viruses [46]. An experiment in which cell cultures were treated with and without IVM (20 μmol/L) over 24 h identified increases in the gene expression of proteins participating in four antiviral pathways that were statistically significant, including the routes of infection of HCMV, HPV, EBV and HIV1. These results support the broad-spectrum antiviral activity of IVM [47]. It is essential to highlight that the movement of proteins between the cytoplasm and the nucleus is mediated by the superfamily of proteins called importins, which are essential for cellular processes, such as differentiation and development, and are fundamental in the pathological states of viral diseases and oncogenesis [48]. The specific viral proteins enter the nucleus of infected cells to perform essential functions as part of the viral replication cycle [49]. An example is the interaction between the HIV-1 integrase protein and the importin α/β1 heterodimer, which is blocked by IVM, thus inhibiting the nuclear import of the integrase protein and, therefore, damaging the viral replication mechanisms [48].

The broad-spectrum antiviral activity of IVM is related to the fact that RNA viruses, to transport viral proteins to the nucleus of the host cell, depend on the importin alpha-beta (IMPα/β1) heterodimer during the viral infection process. This importin is blocked by IVM. The transport of viral proteins through IMPα/β1 to the nucleus occurs in order to inhibit the antiviral response that is assembled by a portion of the host cells. This mechanism has been observed in viruses such as Zika, Dengue, HIV-1, yellow fever, Chikungunya and many more [45,48,50]. Regarding the antiviral response of IVM to DNA viruses, it has been shown that, if the viral proteins necessary for viral replication require entry to the nucleus through IMPα/β1, then this can have an antiviral effect, as in the case of the pseudorabies virus and polyomavirus BK [51].

**Figure 2:**
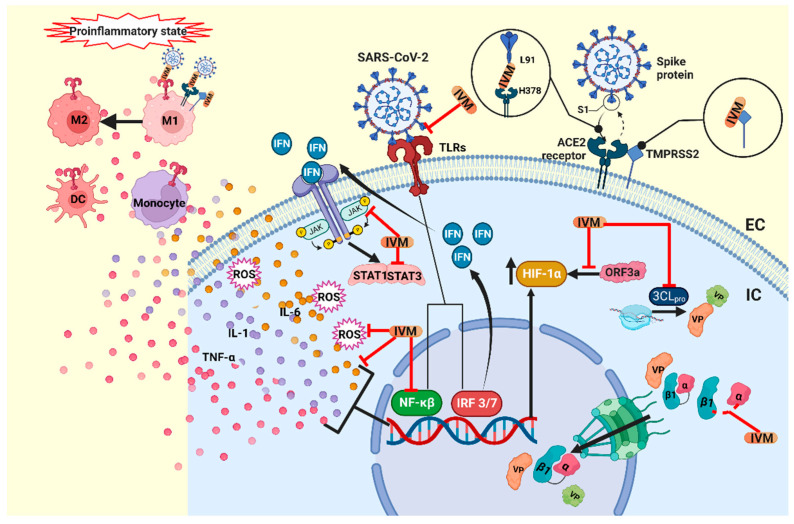
Proposed mechanism of action of ivermectin in COVID-19. IVM blocks the binding complex of the SARS-CoV-2 S protein and the ACE2 receptor, and additionally it blocks the TMPRSS2 protein, inhibiting viral entry into the host cell. IVM could also inhibit the TLR receptors and block NF-κβ, inhibiting the production of the cytokines TNF-α, IL-1 and IL-6 and ROS. TLR also activates IRF3 and IRF7, which initiate the production of type-I and -III IFNs. IFNs activate the JAK/STAT pathway, while IVM can lower the expression of JAK2 and the activity of STAT3. Moreover, in the cytosol, IVM blocks the 3CLpro, the main protease that participates in the viral replication, and blocks the importin complex α/β1 that transports the VP to the nucleus. Furthermore, IVM blocks the overexpression of HIF-1α, induced by the viral protein ORF3a. IVM has also been shown to mitigate the proinflammatory state, where the cytokine storm activates the participation of monocytes, dendritic cells and macrophages, and IVM also promotes the polarization of M2 macrophages over M1. IVM, ivermectin; IC, intracellular; EC, extracellular; VP, viral protein; TLRs, toll-like receptors; NF-κβ, nuclear factor-kappa beta; TNF-α, tumor necrosis factor-alpha; IL-1, interleukin-1, IL-6; interleukin-6; ROS, reactive oxygen species; IRF 3/7, interferon regulatory factors; DC, dendritic cells; M1, M1 macrophage; M2, M2 macrophage; 3CLpro, 3-chymotrypsin-like protease. Created with BioRender.com.

In the case of SARS-CoV-2, it is known that there is no transport of viral proteins to the cell nucleus, as in the case of the infection mechanisms of other viruses. This is because the viral replication cycle takes place exclusively in the cytoplasm of infected cells. However, it is also known that, as part of the antiviral response, there is a communication that involves the transport of proteins related to the regulation of the antiviral responses of infected cells [52,53,54] (Figure 2). There is great controversy regarding antiviral activity in the case of SARS-CoV-2; thus, we believe that more studies are required to clarify the mechanism by which a molecule can be considered to have an antiviral capacity.

One of the first studies to suggest that IVM might have an effect against SARS-CoV-2 reported that IVM caused SARS-CoV-2 viral RNA to be reduced approximately 5000 fold within 48 h of its administration in infected cell cultures [45]. Unfortunately, controversy arose after the publication of this study, after observations that the IVM concentration used in this study was 35 times higher than that approved by the FDA (Food and Drug Administration) for parasitic diseases, which raised doubts about the drug’s efficacy at the FDA-approved doses [55]. It should be noted that the study questioning the dosage was based on an in silico analysis, meaning that the results obtained in an in vivo model could differ. Furthermore, the virus infects the alveolar epithelial cells [55], and in the referred work, the African green monkey kidney cell line, Vero/hSLAM, was used, which does not express the ACE2 receptor, as expressed in the lung tissue.

What is currently known is that one of the mechanisms by which IVM could be effective against SARS-CoV-2 is its interference with the viral entry, since it was shown that IVM interacts with the SARS-CoV-2 spike protein and the ACE2 protein, binding to the spike protein at leucine 91 and the receptor ACE2 at histidine 378 [56,57]. Therefore, it is likely that high doses are not required in order to treat patients with COVID 19, as suggested by some authors. Similarly, Choudhury et al., through in silico studies, indicated that IVM could inhibit the formation of the spike-ACE2 complex formation, targeting the S2 subunit in the spike protein, as well as having a high binding affinity for TMPRSS2, interfering with viral entry. Though these findings are promising, further experimental studies are required to corroborate them [58] (Figure 2).

Through a computational analysis, in which 2447 drugs were analyzed to determine their capacity for interfering with the main protease (3CL pro), which is essential for the replication of SARS-CoV-2, IVM, Diosmin, and Selinexor were identified as candidates for use as anti-COVID-19 drugs through this mechanism of action [59] (Figure 2).

Another option that has been proposed is to enhance the effect of IVM by combining it with other molecules that could enhance its effects. As seen on the Clinicaltrials.gov platform, there are clinical trials of IVM in combination with different molecules. As we can see in Table 1, studies have already published on IVM combinations with nitazoxanide, ribavirin, doxycycline, remdesivir, azithromycin, zinc, aspirin, montelukast, hydroxychloroquine and favipiravir. Out of all the combinations, the general conclusion was that IVM has a synergic effect when used in combination and has a greater effect on symptoms and outcomes.

Some preprints describe therapeutic benefits with safe doses of IVM. Thus, more attention should be paid and research devoted to IVM as a possible antiviral agent against SARS-CoV-2. A clinical trial reported that, after five days of IVM treatment, there was an earlier virological clearance (9.7 days) in the IVM-treated group than in the placebo group (12.7 days) [60]. One study reported that IVM treatment did not affect the viral load of SARS-CoV-2 in the respiratory tracts of infected hamsters and attributed its beneficial effect to its anti-inflammatory effect, as described in the work [61].

### 3.2. Immunomodulatory Effects

An interesting study showed that the standard dose of IVM (400 µg/kg) presented with an immunomodulatory activity through the cholinergic anti-inflammatory pathway, preventing clinical deterioration, reducing the olfactory deficit, and limiting the inflammation of the upper and lower respiratory tract in infected golden hamsters. IN the case of SARS-CoV-2, it was also observed that the IL-6/IL-10 ratio in the lung decreased dramatically [61]. Macrophage polarization towards the M2 subpopulation was observed [61,68]. On the other hand, it was observed that the anti-inflammatory effect is influenced by sex, since the treatment led to a better response in women [61]. It has been suggested that this positive allosteric effect of IVM is caused by the activation of neuronal α7 nicotinic acetylcholine receptors (α-7 nAChR) [69] expressed in the subpopulation of the M2 macrophages [70,71]. Another study performed using a rat spinal cord injury model found that treatment with a combination of IVM and carbon nanotubes led to a decrease in the pro-inflammatory cytokines and oxidative stress modulated by the M1/M2 macrophage subpopulations [72] (Figure 2). Over the past few years, there have been several reports on the anti-inflammatory effects of IVM [73,74], with reports indicating that, to achieve this effect in humans, 36 mg should be administered in a single dose with a standard weight of 70 kg [75].

In in vitro and in vivo models, IVM has been observed to inhibit immune cell recruitment and to suppress mucus hypersecretion and cytokine liberation based on bronchoalveolar lavage in a mouse model of allergic asthma [76]. Similarly, in a model sensitized by lipopolysaccharides (LPS), IVM inhibits the production of TNF-alpha, IL-1 and IL-6 [77]. This effect is probably due to the suppression of pro-inflammatory factors, such as NF-κB and the MAP pathway kinase [74]. Similarly, in an in vitro study, IVM was demonstrated to cause a significant reduction in TNF-α production, induced by TLR agonists, suggesting that IVM could block TLR activity [58,78]. In the pathogenesis of SARS-CoV-2, STAT1 activity is inhibited by the viral proteins NSP1 and ORF6, favoring the activation of STAT3 and enhancing the production of IL-6 [36,79]. IVM decreases the expression of JAK2 [80] and the activity of STAT3 [36,80,81], leading to a reduction in IL-6 production and inflammation. IVM has also been shown to modulate the immune activity in mast cells and macrophages [3] and limit the production of nitric oxide and prostaglandin E2 [82]. Furthermore, in animals infected with SARS-CoV-2, IVM treatment improves clinical outcomes and is associated with a reduction in the inflammatory state, though without impacting the viral load in the upper and lower respiratory tract [61]. Additionally, the effect of IVM is being explored in the context of its participation in the pathogenesis of SARS-CoV-2 [59,83] (Figure 2).

### 3.3. Antitumoral Mechanisms

The effect of IVM as an anti-tumor agent has been explored, and the concentrations necessary for achieving these effects in vivo are within the clinically approved dosages for the treatment of parasitosis [84]. Some of the anti-tumor mechanisms attributed to IVM are the inhibition of the Akt/mTOR and WNT-TCF pathways [85,86], inhibition of MDR proteins, PAK1 helicase, DDX23 and the SIN3 domain [87,88], the activation of the P2X4/P2X7 [89,90], an increment in the chloride channel activity [91], the downregulation of Nanog/Sox2/Oct4 genes [92], and an antimitotic activity (through the damage of tubulin dynamics) [93]. In the breast cancer cell lines MDA-MB-231, MDA-MB-468 and MCF-7, and the ovarian cancer cell line SKOV-3, IVM was demonstrated to have a more significant anti-tumor effect (the induction of the cell cycle arrest at the G_0_-G_1_ phase and reductions in the cell viability and tumor size) and a synergistic effect combined with docetaxel, cyclophosphamide and tamoxifen [86]. In glioma cells, it was observed that it stimulated the activity of caspase-3 and -9, enhancing the expression of p53 and Bax, thus causing apoptosis and blocking the cell cycle in the G_0_/G_1_ phase [94]. On the other hand, IVM increased TFE3-dependent autophagy via ROS signaling pathways in melanoma cells, inducing apoptosis [95]. In porcine trophectoderm and uterine luminal epithelial cells, IVM has also been shown to cause apoptosis through the loss of calcium ion overload, the mitochondrial membrane potential, and the generation of reactive oxygen species [96]. Furthermore, hypoxia, through hypoxia-inducible factors (HIF), plays an essential role in drug resistance [97,98], since, through HIF, cancer cells can resist the decrease in the oxygen concentration and even proliferate. In particular, HIF-1α is translocated to the nucleus by IMPα/β1, and IVM has been shown to block this mechanism [99], making it a viable target for cancer treatments [100]. Furthermore, Tian et al. found that the SARS-CoV-2 protein ORF3a elevates the production of HIF-1α, promoting an inflammatory state, and IVM could potentially mitigate the inflammatory response through the inhibition of HIF-1α [101] (Figure 2).

## 4. Systematic Review of Ivermectin in COVID-19

Despite the many positive outcomes of IVM when used against SARS-CoV-2, just as many studies oppose this statement, leaving us with conflicting perspectives. According to the Clinicaltrials.gov platform, on 18 November 2020, 35 studies were investigating the usefulness of IVM in COVID-19, and almost a year after 30 September 2021, there were 70 studies. To discuss this point, we rely on the systematic reviews and meta-analyses available that have been published to date.

Medical guidelines are generally based on systematic reviews conducted by experts aiming to discern pertinent recommendations for management of the disease. What we can observe in the examination of IVM is that, even though some studies report benefits, when they are analyzed in systematic reviews or meta-analyses, the conclusion reached by most studies is that the evidence is of low quality, with a low level of evidence or with inconclusive data, or even with inconsistencies [83,102,103,104,105,106,107,108].

The main reason for the fact that the WHO does not recommend the use of IVM in patients with COVID-19, except for its use in clinical trials, is that there is a high degree of uncertainty concerning the results, with no clear benefit of its application, in addition to the high risk of bias [30]. However, some studies have reported effects including mortality reduction and clinical improvement [109], as well as the reduction in the length of the hospital stay and better viral clearance [110], while other studies have produced inconclusive data that can neither promote nor refute the efficacy of IVM [111].

In one meta-analysis that included 15 clinical trials, it was reported that IVM reduced the risk of death and that its early use in the clinical course could reduce the number of patients who progressed to severe disease [112]. However, this study was disputed, because it involved a prepress with supposed deficiencies, so that the data was re-analyzed, excluding said study, reaching the initial conclusion once again [112]. Another study described that, when the analysis was limited to patients with mild disease, there was no difference in mortality, but in severely ill patients, the use of IVM significantly reduced mortality [104]. An interesting point to keep in mind is that, in this type of clinical trial, the products of the MDR-1/ABCB1 gene have been reported to influence the entry of IVM into the barrier cells of the gastrointestinal system, and patients with polymorphisms of this gene should be excluded from these studies [67]. Polymorphisms in this gene could partially explain the suboptimal responses to IVM in some studies [113]. Another point to consider is that of comorbidities, since they have been observed to influence the response to IVM, as in one study where it was determined that hypertension decreased the benefits of IVM [114].

Moreover, Elgazzar et al. conducted a study comparing 6 groups of 100 patients each, with group I and III given an IVM + SOC treatment, applied to mild/moderate and severe cases respectively; group II and IV were given a hydroxychloroquine + SOC treatment, applied to mild/moderate and severe cases respectively; and healthcare workers (HCWs) and household contacts were divided into groups V and VI, who were given IVM and personal protective measures (PPM), including a prophylaxis vs. only PPM, respectively. The patients from groups I and III showed statistically significant clinical improvement and a reduction in their mortality rates compared with groups II and IV, and in group V the incidence of infection was reduced compared with group VI, indicating that IVM was effective not only as a coadjutant drug, but as a prophylactic as well [115]. In another study, where the prophylactic capacity of IVM was explored, 131 HCWs were treated with a combination of topical IVM combined with carrageenan (IVER.CAR) compared with 98 subjects without treatment. Over the span of the 28 days of the study, none of the HCWs in the IVER.CAR group tested positive for SARS-CoV-2 according to PCR tests compared to 11.2% of cases in the group treated without IVER.CAR [116].

On the other hand, Marcolino et al. reviewed, in a meta-analysis, 25 randomized control trials assessing clinical outcomes in COVID-19 patients treated with ivermectin compared to a group treated with a placebo and standard of care (SOC) treatment, concluding that IVM did not reduce the risk of mortality (RR = 0.76; 95%) or risk of the need for mechanical ventilation (RR = 0.74; 95), although no added risk or adverse effects were reported [117]. There is evidence from one study showing that, at the standard doses, IVM treatment did not have a significant impact on clinical or microbiological outcomes compared with the SOC group, although less patients in the IVM group required intensive care compared with those in the SOC group (38% vs. 69% respectively) [118]. Similar findings were reported in other studies, which did not observe any significant differences in the viral load, outcomes or adverse events in the IVM groups treated with a standard dosage [119,120], although higher IVM plasma levels were correlated with a decrease in the viral load in a dose–response manner [120]. Although the safety profile of IVM at higher doses is comparable to that of the standard doses [121], there exists a concern that the doses required to reach clinically effective levels are not feasibly safe (10× higher in order to reach the IC50). It should be noted that this was an in silico study, which should be corroborated by experimental studies [55].

## 5. Ivermectin in COVID-19 Comorbidities

### 5.1. Nosocomial Pneumonia

Bacterial coinfections are common in respiratory viral infections [122,123], and patients with COVID-19 are no exception [124]. Of the various studies that report on this situation, only a few representative studies are mentioned here. One study reported that, of 340 COVID-19 patients, 12% had secondary bacterial infections, and of these, 25.59% belonged to the species Klebsiella, 20.93% to methicillin-sensitive Staphylococcus aureus, 16.28% to Escherichia coli, 13.95% to methicillin-resistant Staphylococcus aureus, 11.63% to Enterobacter, 2.32% to Streptococcus pneumonia and 9.30% to Pseudomonas aeruginosa. Of the Enterobacteriaceae isolates, 74% were resistant to cotrimoxazole, 67% to piperacillin, 47.5% to ceftazidime and 42% to cefepime [123].

It should be noted that atypical bacteria (Mycoplasma pneumoniae, Chlamydia pneumoniae, and Legionella pneumophila) may be masked by the presentation of COVID-19, as they have overlapping clinical and imaging features, and the timely identification of this co-infection could be vital in critically ill patients [125]. Severely and critically ill patients are especially susceptible to co-infections. In one study, serum fungal antigens were observed more frequently in the critical group than in the severe group, and the positive frequency rate of serum fungal antigens increased with a prolonged stay in the intensive care unit (ICU) [126]. These findings were replicated in seven ICUs in England, where an increase in the proportion of pathogens was correlated with the length of stay in the ICU, with the identification of mainly Gram-negative bacteria, particularly Klebsiella pneumonia and Escherichia coli. Patients with co-infections/co-colonization were more likely to die in the ICU than those without co-infections [127].

Many factors can influence the development of co-infections in terms of the frequency and type of pathogens, including the level of development of the country or region—such is the case of COVID-associated Mucormycosis in India [128]. Some studies even agree that antibiotic therapies targeting respiratory pathogens should be considered in severe cases [129]. However, there developed a growing concern during the pandemic that the widespread use of empirical antibiotics could contribute to the rise of multidrug-resistant microorganisms, and antimicrobial administration programs are required to minimize and reduce this threat [130]. Although, in theory, antibiotics do not directly affect SARS-CoV-2, viral respiratory infections often result in bacterial pneumonia. Some patients may die from bacterial coinfection rather than the viral infection itself; therefore, bacterial coinfections are considered critical risk factors for COVID-19 severity and mortality [131]. Inversely, a predictor of rapid recovery from COVID-19 is the absence of bacterial coinfections [132].

Some antibiotics are obtained from fermentation carried out by Gram-positive bacteria of the genus Streptomyces, as in the case of Streptomyces griseus, from which the well-known streptomycin is derived. Therefore, it is not surprising that the fermentation products of Streptomyces avermitilis have antibiotic properties. Strategies have been proposed that aim to search for new antimicrobials in order to combat multidrug resistance, and the repurposing of IVM as an antibiotic has potential. Among the avermectins group, IVM stands out for its antibacterial effects. In clinical isolates of multidrug-resistant Mycobacterium tuberculosis, IVM has shown bactericidal effects [133]. Additionally, avermectins such as doramectin, IVM, moxidectin and selamectin inhibit the growth of strains of Mycobacterium Bovis BCG, Mycobacterium tuberculosis from H37Rv, CDC 1551, Erdman, and Mycobacterium smegmatis at concentrations ranging from 1 to 8 μg/mL [133]. It has also been observed to have an antibacterial effect against Staphylococcus aureus at concentrations of 6.25 and 12.5 μg/mL [134]. Macrolide antibiotics have a distinctive macrocyclic lactone ring, and their mechanism of action works through the inhibition of bacterial protein synthesis. However, they also have modulatory effects on the host defense responses and inflammatory responses [135]. An example of this is the activation of the P2X4 receptors by IVM in macrophages, increasing the destruction of bacteria and protecting against sepsis [136], which is most likely the most prominent antibacterial effect of IVM.

### 5.2. Wound Healing

Many COVID-19 patients show symptoms of acute lung injury that can eventually lead to pulmonary fibrosis [137]. The treatment of inflammation with corticosteroids reduces inflammation and the likelihood of developing fibrosis [138]. Regarding the effect of IVM on wound healing, a study reported that IVM cream, at low dosages (0.03–0.1%), induced wound healing, with minimal scarring, and decreased the macroscopic indices of wounds, such as exudation, the edge of oedema, hyperemia and granulation tissue deposits [139]. Other works report a decrease in skin inflammation under certain conditions [140,141,142,143], which can help to avoid scar formation. It would be interesting to explore this mechanism of IVM directed against post-COVID-19 pulmonary fibrosis.

## 6. Discussion and Conclusions

The rush to obtain potential drugs for the treatment of COVID-19 patients has led to an array of studies on IVM of varied qualities and even methodological questioning. The efficacy of IVM in human SARS-CoV-2 infection is still under investigation. The authors of most meta-analyses agree that controlled, randomized, placebo, double-blind and sufficiently powered trials are required to obtain a definitive conclusion, and this requires a large enough number of subjects and robust experimental designs. It is difficult to believe that a single molecule can have effects as diverse as those described here. One of the most surprising studies is the one carried out at the Pasteur Institute in France, in which the authors conclusively proved the drug’s anti-inflammatory effect [61]. Another prominent effect is the stimulation of the bactericidal effect on the immune cells, which is of particular benefit in the management of bacterial coinfections in COVID-19 patients, especially in severe and critical patients who require ICU admission or experience prolonged hospital stays and are at risk of nosocomial bacterial infections. This is probably, in part, responsible for the favorable effect of IVM observed in some clinical trials.

IVM could also potentially benefit pulmonary fibrosis patients with PASC. The results observed in wound healing treated with IVM, attributed to its anti-inflammatory effects or inhibition of the nuclear translocation of HIF-1α, make it a promising antifibrotic agent. Therefore, we propose that future research should explore these mechanisms of IVM in detail in experimental in vivo and in vitro models. Interestingly, the anticancer effect of inhibiting HIFs raises the possibility of the drug’s potential use in antitumor therapies, especially since the inhibition of the nuclear translocation of HIF-1α could mitigate drug resistance. Evidence of all these effects is still being developed, but if they are demonstrated, IVM could be effective for treating various diseases. Most studies performed on IVM in COVID-19 patients have focused on the proposed antiviral effects; however, the clinical effects of IVM in these patients could be achieved through the added participation of multiple mechanisms of action that are not limited to its antiviral activity.

To conclude, owing to the vast assortment of possible therapeutic targets of IVM, including the direct targeting of the antiviral machinery of SARS-CoV-2, as well as the pro-inflammatory state it induces, including the cytokine storm, the potential of this drug is promising to say the least. The complexity of the pathogenesis of COVID-19 has led to divergences in the clinical application of this drug, with the possibilities for its use ranging from the prophylactic state all the way up to the treatment of PASC. For the sake of reaching a consensus on the therapeutic efficacy of IVM and shed light on the mechanisms of action of this drug, further experimental and clinical studies should be considered, with greater standardization in the treatment regimen.

Due to the immense level of public interest, the literature on the effects of IVM in COVID-19 is of highly variable quality, with several large studies, with a good degree of confidence [107,144] and questionable credibility, suggesting that the drug could save lives, a claim that later turned out to be untrue, and many studies that have not been properly peer reviewed [112]. Furthermore, pharmacologically, it is not possible to safely reach the plasmatic levels required for the proposed mechanisms of action to prevent the SARS-CoV-2 infection in vitro or to function as a 3CL protease inhibitor. Additionally, IVM is easily available to the public, and though many patients have proper medical requirements validated through medical prescriptions, other opt to self-medicate, meaning that they are at risk of side effects or improper dosages. Moreover, IVM is frequently used as a veterinary drug, and it could be (and has been) misused by the public, which has been addressed in an FDA statement. While we acknowledge the many beneficial effects of ivermectin, which has saved countless lives as an anti-parasitic agent, even though the proposed mechanisms directed against the COVID-19 infection are promising, they are still largely inconclusive and require further study in order to elucidate whether the drug’s application in the treatment of these patients will be beneficial.

## Figures and Tables

**Table 1:** **Clinical and experimental effectiveness of combining Ivermectin with other drugs in COVID-19.** Studies already published on IVM combinations with other drugs.

Study Population	Combination IVM with:	Results
Sixty-two patients on a triple combination therapy versus fifty-one patients on symptomatic supportive therapy matched for age and sex.	Nitazoxanide and Ribavirin compared to routine supportive treatment.	This study showed that the clearance rates were 58.1% and 0% on day 7 and 73.1% and 13.7% on day 15 in the combined antiviral group compared to the symptomatic support treatment group. Therefore, the combined use of nitazoxanide, ribavirin and ivermectin plus a zinc supplement effectively eliminated SARS-CoV2 from the nasopharynx in a shorter time than symptomatic therapy [57].
Two hundred patients with mild to moderate symptoms of COVID-19 were randomly assigned to the treatment group and two hundred to the placebo group.	Doxycycline versus placebo.	The median time to recovery was seven days (4–10) in the treatment group and 9 (5–12) in the placebo group, while the percentage of patients with a recovery of ≤7 days was 61% and 44%, respectively [62].
In vitro model of RAW264.7 macrophages infected with MHV.	Remdesivir.	The combination of remdesivir and ivermectin showed a highly potent synergism by significantly reducing the 7-log10 of live virus and 2.5-log10 of viral RNA in infected macrophages. This combination also resulted in the lowest IL-6, TNF-a and leukemia inhibitory factors [63].
The intervention group of five hundred and eighty-five patients and control group of five hundred eighty-five patients were treated with a placebo, along with a second control group of one hundred and thirty-seven untreated patients.	Azithromycin plus nitazoxanide or hydroxychloroquine.	Compared with control group 1 and control group 2, the intervention group showed a 31.5 to 36.5% reduction in viral excretion (*p* < 0.0001), 70 to 85% in the duration of symptoms (*p* < 0.0001) and 100% in respiratory complications, hospitalization, mechanical ventilation, deaths and post-COVID manifestations (*p* < 0.0001). For every 1000 confirmed cases of COVID-19, at least 70 hospitalizations, 50 mechanical ventilation and 5 deaths were averted [64].
Four hundred and eighty-one patients with combined therapy and two hundred and eighty-seven with standard treatment.	Azithromycin, montelukast, and acetylsalicylic acid vs. standard therapy.	A total of 85% of cases who received the combined therapy recovered within 14 days, and the total was 59% in the comparison group. The likelihood of recovery within 14 days was 3.4 times greater among the combined therapy group than in the comparison group. Patients treated with the combined therapy had a 75% and 81% lower risk of being hospitalized and death, respectively, than the comparison group [65].
Nine hundred and twenty-two outpatients, of which three hundred and twenty were given a multidrug therapy with ivermectin.	At least two agents with antiviral activity against SARS-CoV-2 (zinc, hydroxychloroquine) and one antibiotic (azithromycin, doxycycline, ceftriaxone).	A total of 320/922 (34.7%) patients were treated, resulting in 6/320 (1.9%) and 1/320 (0.3%) patients hospitalized and who died, respectively. We concluded that early ambulatory (not hospitalized, treated at home) multidrug therapy is safe, feasible and associated with low rates of hospitalization and death [66].
Sixty-six patients were included in the study, with thirty-six in the study group and thirty in the control group.	Reference treatment protocol: hydroxychloroquine + favipiravir + azithromycin. Patients in the control group received only standard treatment with three other drugs, without ivermectin.	At the end of the first 5-day follow-up period, the rate of clinical improvement was 73.3% (22/30) in the study group and 53.3% (16/30) in the control group (*p* = 0.10). At the end of the follow-up period, the mean peripheral capillary oxygen saturation (SpO_2_) values of the study and control groups were 93.5 and 93.0%, respectively. PaO_2_/FiO_2_ ratios were determined as 236.3 ± 85.7 and 220.8 ± 127.3 in the study and control groups, respectively. At the end of the follow-up period, mortality was recorded for 6 patients (20%) in the study group and 9 (30%) patients in the control group (*p* = 0.37) [67].

## Data Availability

Not applicable.
